# Check-Rein Technique for Management of Neglected Locked Posterior Shoulder Dislocations: Evaluation of Mid-term Outcome of a Novel Technique

**DOI:** 10.5704/MOJ.1611.003

**Published:** 2016-11

**Authors:** NK Magu, P Gogna, A Singh, R Rohilla

**Affiliations:** Department of Orthopaedics, Pt BD Sharma, Rohtak, India

**Keywords:** Neglected locked posterior dislocation, shoulder dislocation, coracoid

## Abstract

**Introduction:** Neglected locked posterior shoulder dislocations, although rare, are quiet perplexing to manage. Various treatment methods have been explained for their management, but a consensus is still lacking. Besides describing a novel technique for the management of these lesions, this study aims to evaluate the mid-term outcome of this technique.

**Method:** This prospective study involved seven consecutive patients with locked posterior dislocation of the shoulder with humeral defect between 25% and 50%. All patients underwent open reduction of the locked posterior dislocation with the current technique. The final outcome was assessed at a mean follow up of 3.5 years (range 2-5 years) using the DASH score.

**Result:** The mean age of the patients was 32 years (range 21-44) and all were men. The mean time to presentation from initial injury was 2.4 years (range 2-4 years). The patient related outcome as measured by DASH score improved from a preoperative mean of 59.1 to mean value of 8.6 at the time of final follow up. There were no cases of graft pull out, nonunion at the graft site or infection.

**Conclusion:** This technique results in pain-free range of motion with a stable shoulder though a larger sample population with a longer follow up is required to further support our observations.

## Introduction

Treatment of chronic posterior shoulder dislocation depends upon the size of the humeral head defect. For defects of less than 25% of the humeral head articular surface, grafting of the defect is seldom necessary, while for defects larger than 50% of the articular surface, shoulder arthroplasty is advocated. The defects between 25% and 50% of the humeral head of the humerus present a demanding situation with no strict treatment guidelines^1-5^. The goal remains to reduce the head and graft the engaging reverse Hill-Sachs lesion so that further locking can be prevented^6^. A number of procedures have been described to tackle this lesion, however invariably all of them have their own drawbacks, which makes it difficult to lay standard guidelines for management of these lesions. There is need for a newer technique, which deals with the pathology efficiently, and is free from the drawbacks of the currently used techniques. We describe our experience with a novel technique in the management of these quandrous lesions, by doing coracoid osteotomy and using it for filling the humeral head defect. To the best of our knowledge, this technique has not been described in the English literature.

## Materials and Methods

This prospective study involved seven consecutive patients with locked posterior dislocation of the shoulder between 2005 and 2013. While six patients were known epileptics and sustained these lesions secondary to seizures, one of them gave a history of trauma secondary to road traffic accident in which he sustained head injury as well and the shoulder dislocation went neglected. The study was performed after institutional ethical clearance and consent was obtained from patients to include them in the study. Patients were clinically examined for any associated neurological injuries. All the patients underwent preoperative radiological study including plain radiographs and CT scans to confirm the diagnosis and assess the defect size in the humeral head. Only those patients in whom the humeral defect was between 25 and 50 percent were included in the study. The patients underwent surgery under general anaesthesia after informed consent was obtained.

The standard deltopectoral approach to shoulder was used^6^. The anterior part of the deltoid muscle was retracted laterally to expose the structures around the coracoid process and the anterior part of the joint capsule. To have a better view of the deep aspects of the shoulder, including the anterior margin of the glenoid, the coracoid process was osteotomized. The periosteum of the superior aspect of the coracoid was incised followed by osteotomy through the bone. The osteotomised coracoid process along with the conjoint tendon (coracobrachialis and the short head of biceps) was then reflected medially and distally. The subscapularis at its musculotendinous junction about 2.5 cm medial to its insertion into the lesser humeral tuberosity was divided. This gave a wide view to the shoulder joint including the Hill-Sachs lesion and the glenoid. The shoulder joint was reduced under vision, with a gentle lateral thrust and external rotation. If reduction was not facilitated by this maneuver, we used a Cobbs elevator paying attention not to further damage the humeral head and glenoid. The joint was then thoroughly irrigated. To restore the shape of the humeral head, the osteotomized coracoid was pulled in through a rent made in the subscapularis and fixed onto the defect in the anterior aspect of humeral head using one or two screws so as to acts as a check rein to posterior dislocation of the shoulder, and that is why we named this technique as “check-rein technique”. The subscapularis was then repaired, and the wound was closed over suction drain. The stability of the shoulder and the construct were assessed intraoperatively under direct vision and image intensifier.

Postoperatively, the shoulder was immobilized with an external rotation brace for six weeks. Physiotherapy including passive, active-assisted, and progressively active range of motion and rotator cuff strengthening exercises were started at six weeks, gradually progressing to full activity at 12 weeks. The final outcome was assessed as per the DASH score.

## Result

The mean age of the patients was 32 years (range 21-44 years) and all were men. The mean time to presentation from initial injury was 1.4 years (range 1-3 years). At the time of final follow up of 3.5 years (range 2-5 years) all patents had a satisfactory outcome (Fig. 3). The patient related outcome as measured by DASH score improved from a preoperative mean of 59.1 to postoperative mean value of 8.6. The shoulders which were previously locked were able to regain a pain-free range of motion, with gradual physiotherapy, and none of the patients developed any further episode of dislocation after the procedure. There were no cases of graft pull out, nonunion at the graft site or infection.

**Fig. 1:**
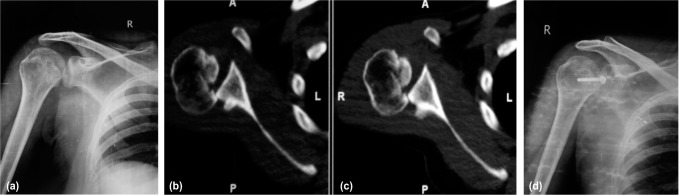
(a) Radiographs of a 21-year old male showing posterior dislocation of the shoulder. It was neglected and patient presented to us at 5 months post injury. (b) CT scans showing engaging reverse Hill-Sachs lesion. (c) Radiographs of the same patient post surgery at 2 years follow up.

**Fig. 2:**
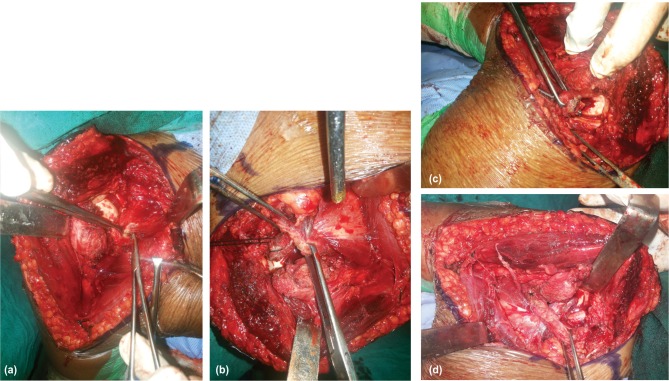
(a) After open reduction of the shoulder using delto-pectoral approach a rent was made in the subscapularis though which the osteotomised coracoid with its attachments was withdrawn. (b) Image showing coracoid being withdrawn through the rent in subscapularis. (c) The osteotomised coracoid along with its attachments was fixed onto the humeral head defect. (d) The coracoid with its attachments has been fixed in the humeral head defect.

**Fig. 3:**
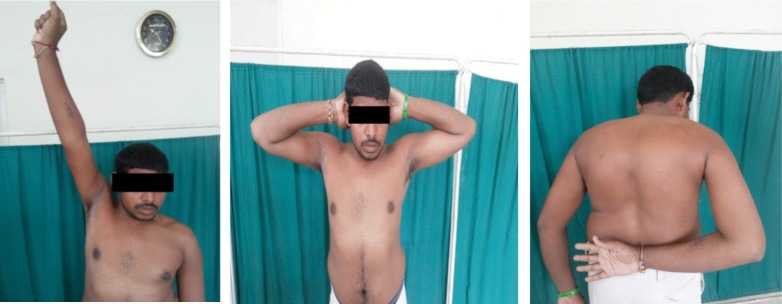
a-c Images showing good post-operative range of motion at one year follow up.

## Discussions

In chronic posterior dislocations, a bony lesion similar to the Hill-Sachs lesion of recurring anterior dislocations is found which is named “Reverse Hill-Sachs lesion”. It results from compression fracture of the anteriomedial aspect of humeral head due to impingement by the posterior rim of the glenoid^6^. These lesions tend to increase in size with chronicity, attributable to patient’s continual attempts to increase the range of motion of the affected locked joint. During open reduction, it is essential to prevent recurrent dislocations caused by the engaging Hill-Sachs lesion^6^.

The Hill-Sachs lesion of posterior shoulder dislocation with defect size 25% to 50% mandates grafting and humeral head reconstruction^1-4^. McLaughlin in 1952 first described subscapularis tendon transfer at the site of humeral head defect in patients with posterior shoulder dislocation^7^. Neer described the technique of transplanting the subscapularis tendon with the lesser tuberosity attached on the grounds that bone-to-bone healing is better than tendon to bone healing^8^. Delcogliano *et al* treated four patients by the McLaughlin technique and the Neer modification (one with posterior bone block), and all had acceptable results^9^. Charalambous *et al*^1^ modification of the McLaughlin technique included plication of the subscapularis tendon into the humeral head defect using suture anchors^10^. These techniques involved either sacrificing the prime internal rotator of the shoulder, or doing a new osteotomy of the lesser tuberosity, adding on to the morbidity. These techniques although gained popularity, had the drawback of restriction of internal rotation and concern for possible subsequent shoulder replacement.

Anatomical reconstruction of the humeral head gained popularity with Gerber describing autogenous iliac crest bone grafting for the defect as a successful technique for acute traumatic dislocations^11,12^. Later, Dubousset and Lambert suggested combined reconstruction of the shape of the humeral head with autogenous iliac crest bone graft and posterior capsulolabral complex^13^. However, harvesting bone grafts adds to the surgical time and is associated with donor site morbidity. Rotation osteotomy of the humerus was described by Chaudhari *et al*for recurrent dislocation of the shoulder^14^. In their study of ten patients with locked posterior dislocation of the shoulder, managed with allograft reconstruction of the humeral head and rotational osteotomy of the proximal humerus, Keppler *et al* reported six patients with good results, two fair and two poor. Both the patients with poor results had advanced articular cartilage damage^15^. However, the latter technique did not gain popularity due to technical difficulties and risk of devascularization of the humeral head^5^. Moreover, tissue matched donor bone is not readily available at many centres worldwide especially in developing countries. There are also issues with disease transmission.

We found the current technique to be more suitable than McLaughlin’s technique and its later modification in several ways. Firstly, the osteotomy of the coracoid gave a wide view to the shoulder joint which aided in disengaging the humeral head and reducing the shoulder joint. We found it to be better than autologous iliac grafting as it prevented donor site morbidity. Also, the graft, being a muscle pedicle bone graft, instead of cortical or cortico-cancellous bone graft incorporates itself well. The coracoid with its attachments routed through a rent in subscapularis and inserted onto the humeral head defect acts as a check rein to posterior dislocation of the shoulder.

The limitation of our technique is that it is a non-anatomical reconstruction procedure, making subsequent shoulder arthroplasty difficult. Though this technique resulted in pain-free range of motion, a stable shoulder, and good joint congruency in our patients, a larger sample population with a longer follow up is required to further support our observations.
